# Supplementary material for: Modulation of Titin and Contraction-Regulating Proteins in a Rat Model of Heart Failure with Preserved Ejection Fraction: Limb vs. Diaphragmatic Muscle
Source: Int J Mol Sci. 2024 Jun 16;25(12):6618. doi: 10.3390/ijms25126618 (PMC11203682; doi:10.3390/ijms25126618)
Supplement: Supplementary file 1 [file ijms-25-06618-s001.zip › ijms-3049702-supplementary.pdf]

Sarcomere organization

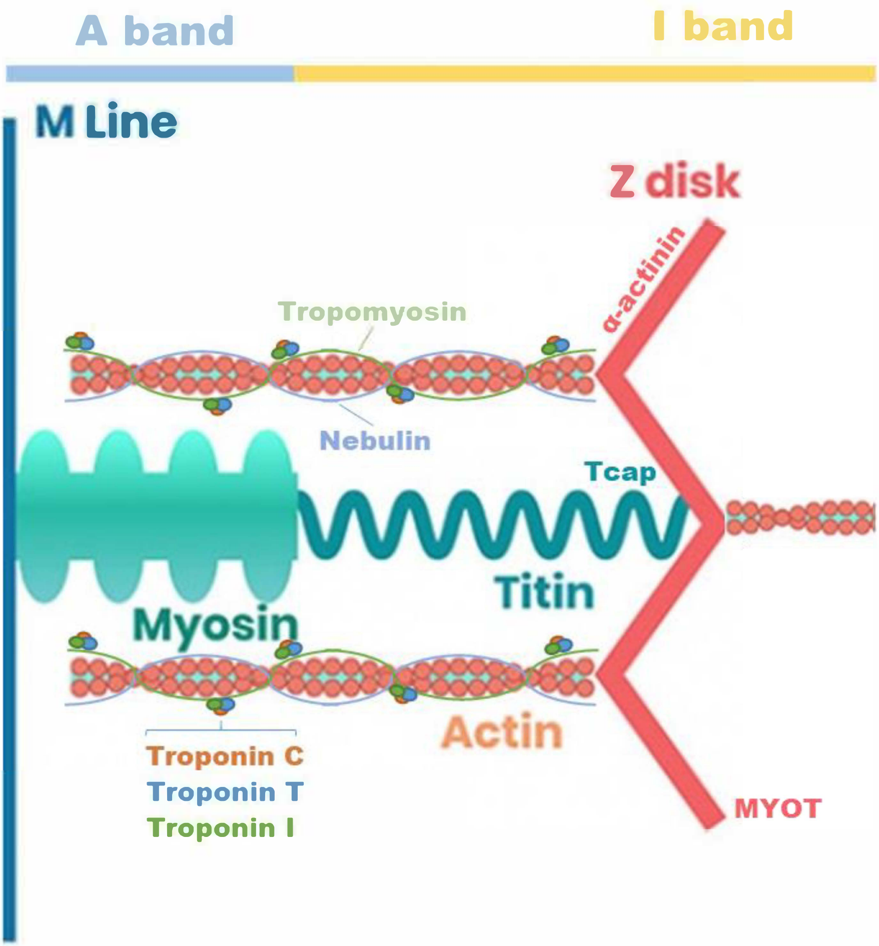

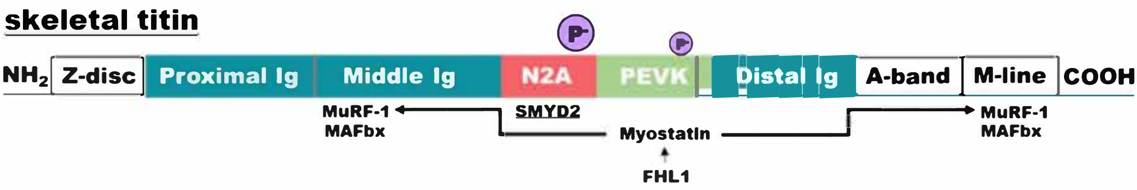

Supplemental Figure S1 – sarcomere organization regarding the examined proteins localization and full-length skeletal titin in an healthy organism
